# New Green Determination of Cu, Fe, Mn, and Zn in Beetroot Juices along with Their Chemical Fractionation by Solid-Phase Extraction

**DOI:** 10.3390/molecules24203645

**Published:** 2019-10-09

**Authors:** Pawel Pohl, Mateusz Pieprz, Anna Dzimitrowicz, Piotr Jamroz, Anna Szymczycha-Madeja, Maja Welna

**Affiliations:** Division of Analytical Chemistry and Chemical Metallurgy, Faculty of Chemistry, Wroclaw University of Science and Technology, Wybrzeze Wyspianskiego 27, 50-370 Wroclaw, Poland; pieprzmateusz1997@gmail.com (M.P.); anna.dzimitrowicz@pwr.edu.pl (A.D.); piotr.jamroz@pwr.edu.pl (P.J.); anna.szymczycha-madeja@pwr.edu.pl (A.S.-M.); maja.welna@pwr.edu.pl (M.W.)

**Keywords:** beetroot juice, microelements, flame atomic absorption spectrometry, sample preparation, solid-phase extraction, chemical fractionation

## Abstract

A new simple and rapid method for the determination of the total concentrations of Cu, Fe, Mn, and Zn in beetroot juices by flame atomic absorption spectrometry was developed and validated. The method included a very simple sample preparation, i.e., the two-fold dilution and acidification of the samples with HNO_3_ to 1 mol·L^−1^ and provided the precision within 2–3% and the trueness better than 6%. The method was applied for the rapid screening analysis of the different commercially available beetroot juices. The chemical fractionation of Cu, Fe, Mn, and Zn was also proposed by the two-column solid-phase extraction with the reversed-phase and cation exchange tubes. It was revealed that Cu, Fe, Mn, and Zn were primarily in beetroot juices in the form of the organically bound forms, contributing to the distinguished hydrophobic and residual fractions. The sums of the mean contributions of both fractions were up to 98% (Cu, Fe, Zn) and 100% (Mn), pointing out that no labile nor unbound forms of the studied metals were present in the matrix of beetroot juices.

## 1. Introduction

The consumption of fresh organic vegetables or juices prepared from them has been increasing incredibly fast in recent years and now it occupies an important place in the daily diet of those who practice sports, exercise, and other activities [1]. The attractiveness of vegetable juices particularly continues to grow among the different age groups of the citizens of the European countries because of their high health awareness and a tendency to avoid or eliminate fruit juices and fruit beverages due to the high sugar content of the fruits themselves. According to the Centre for the Promotion of Imports from the developing countries (CBI) [2], the major importing and consuming countries of the vegetable juices are Belgium, the Netherlands, the United Kingdom, France, and Germany, where food authorities highly recommend high-value green and red colored vegetable juices [2]. Beetroots and beetroot juices (BRJs) occupy a special place among other vegetables in the daily diet of the athletes and people practicing the endurance sports and activities [3]. They have the desirably low contents of fructose and high contents of sucrose, hence, their intake can naturally boost the energy levels and exercise capacity [3,4]. The presence of betalains, i.e., betacyanins and betaxanthins, in these vegetables and fresh juices made from them, in addition to the naturally occurring nitrates, has a key role in preventing the cardiovascular diseases because these species helps in improving the blood sugar level and lowering hypertension [5]. Beetroots and their juices are recognized as well to contain one of the highest amounts of the phenolic compounds amongst other vegetables, hence, their antioxidant and antiradical activity is obvious, and therefore, incorporating them in the daily diet is highly beneficial [6,7].

Beetroots additionally have one of the highest concentrations of several macroelements, namely K, Na, Ca, and Mg (in the order of the highest concentration), as well as the microelements (Fe, Mn, Zn, Cu), even higher than found in other vegetables, e.g., carrots or spinach [7,8,9]. In this case, however, the health-promoting effects of beetroots result not only from the intake of high amounts of the mentioned macro- and microelements but primarily from the beneficial concentration ratios of selected minerals in these vegetables, particularly K to Mg, Ca to Mg, and K to the sum of Mg and Ca [1]. In reference to this, the elemental analysis of beetroots by the atomic spectrometry methods has to be carried out to possess the crucial information on their mineral composition, the levels of the accumulated trace metals that could pose a threat to their quality and safety, and any changes in the concentrations of the selected metals due to the applied cultivation methods or cultivars [1,8,10,11,12,13,14].

Surprisingly, very few scientific works have been published so far on the elemental analysis of BRJs [1,4]. The information about the mineral composition of BRJs appears to be of special nutritional concern because it may give the answer about the health benefits of these more and more popular vegetable beverages, particularly in reference to the present microelements like Cu, Fe, Mn, and Zn. In this case, one can expect that the rapid and simple green analytical method for the elemental analysis of the BRJs will certainly help in very quickly and unambiguously assessing their quality and safety declared by the producers. The information that they do not contain any unacceptable concentrations of the selected microelements or trace metals at the stage of their handling, preparation, and storage is very advantageous and can quickly be provided only with such newly developed and validated green analytical methods.

To fill the gap associated with the lack of the appropriate green analytical methods for the elemental analysis of BRJs, the main objective of the present work was to develop a simple and fast flame atomic absorption spectrometry (FAAS) method for determining the selected nutritionally essential microelements, i.e., Cu, Fe, Mn, and Zn, in these vegetable beverages. The research hypothesis taken for this study assumed that it would be possible to in situ decompose the sample matrix components in an air-acetylene flame of FAAS, and release Cu, Fe, Mn, and Zn from their bound forms prior to the quantitative and interference-free measurements by only appropriately diluting the samples of BRJs and acidifying them with HNO_3_ to a proper concentration. The green analytical method developed in this work was then validated and applied for the analysis of several commercially available BRJs. Additionally, the operationally defined speciation by using the two-column solid-phase extraction (SPE) with the reversed-phase and cation exchange sorptive materials was used to get an insight into the distribution of total Cu, Fe, Mn, and Zn among different bound and unbound speciation forms, varying due to the hydrophobicity and charge, hence, having the dissimilar lability, and bioavailability. Accordingly, three operationally defined fractions of the studied metals, i.e., the hydrophobic fraction (HF), the residual fraction (RF), and the cationic fraction (CF), were distinguished and determined in the selected BRJs.

## 2. Results and Discussion

### 2.1. Evaluation of the Reference Method

To assess the ability of several alternative, non-digestive sample preparation procedures involved in the very simple and rapid FAAS-based method for the elemental analysis (Cu, Fe, Mn, and Zn) of BRJs, it was necessary to determine the bias that such method could generate. Unfortunately, no adequate certified reference material for the elemental analysis of BRJs is available until now. According to the recommendation of the ISO Standard no. 5725-4:1994 [15], the results obtained with the tested alternative sample preparation procedures followed by the FAAS analysis of the prepared sample solutions were referred to the results obtained with the reference method, acknowledged to gain the reliable concentrations of Cu, Fe, Mn, and Zn in BRJs. It was previously approved that such reference method (the reference method no. 1, RM1) was wet digestion with concentrated HNO_3_ in a digestion block, aimed at decomposing the organic matrix of BRJs in the temperature of 130 °C, and releasing Cu, Fe, Mn, and Zn in the form of their simple ions, followed by the FAAS analysis of the resulting sample solutions. The validity of the results achieved with this reference method was verified by comparing them with the results of the analysis of the same samples but obtained with the aid of the method that was based on different physicochemical properties than FAAS [16]. In this case, the samples were decomposed in concentrated HNO_3_ in the digestion block as well, while inductively coupled plasma optical emission spectrometry (ICP OES) was used to analyze the resulting sample solutions for the content of Cu, Fe, Mn, and Zn (the reference method no. 2, RM2). In all these initial experiments, BRJ5 was used.

Before the average concentrations of Cu, Fe, Mn, and Zn determined with both reference methods were compared, the F-test was conducted to test the assumption about the homogeneity of the variances of both sets of the results, expressed as the standard deviations (SDs) [17]. It was established that the calculated values of this test (F_calculated_) were lower than its critical value (F_critical_ = 5.391, α = 0.05, df_1_ = 3, df_2_ = 3) for all the determined metals (see the respective F_calculated_ values in Table 1). This pointed out that the variances of both sets of the results were statistically identical, hence, the independent two-sample t-test was used to compare the average concentrations of Cu, Fe, Mn, and Zn obtained with both reference methods [17].

Again, no statistical differences between the concentrations of Cu, Fe, Mg, and Zn measured with both reference methods, i.e., wet digestion with concentrated HNO_3_ in the digestion block along with the measurements of the resulting sample solutions by FAAS or ICP OES, were found (see the respective t_calculated_ values in Table 1). The calculated values of the applied t-test were lower than its critical value (t_critical_ = 2.447, α = 0.05, df = 6). This confirmed that the FAAS-based reference method (RM1) provided reliable results of the analysis for all the studied metals here in the matrix of BRJs and hence it was used in all further experiments.

### 2.2. Alternative Sample Preparation of Beetroot Juices Prior to Their Simple and Rapid FAAS Analysis

At the stage of the development of the alternative sample preparation procedures prior to the FAAS analysis of the BRJs, the effect of no-treatment of the respective samples (direct analysis) or their dilution (×5, ×3, and ×2) with water, along or not with their acidification with HNO_3_ to 0.5 or 1 mol L^−1^, was studied in relation to the reference values for Cu, Fe, Mn, and Zn. The final concentrations of HNO_3_ in the prepared sample solutions were particularly selected not to provoke any additional non-spectral interference from the added acid. It was expected that the acidification of the samples of the BRJs would likely result in releasing Cu, Fe, Mn, and Zn from their bound forms, ensuring the in situ “ashing” and atomization of their organic compounds [18]. 

The results obtained for Cu, Fe, Mn, and Zn for the complete set of the combinations of the alternative sample preparations with the FAAS analysis of the resulting sample solutions are given in Table 1. As before, the F-test (at α = 0.05, df_1_ = 3, df_2_ = 3) was used to compare the SDs achieved for the set of the results obtained using the simplified methods (SM1–SM10) with those for the results obtained using the reference method no. 1 (RM1). Upon the outcomes of this test, it was verified whether the use of the two-sample t-test for the comparison of the average concentrations of Cu, Fe, Mn, and Zn would be justified. Since no statistically significant differences between the compared SDs were found (the respective F_calculated_ values were lower than the F_critical_ value), the homogeneity of the variances for the compared results was confirmed, and hence, the t-test was appropriate to compare the average concentrations of Cu, Fe, Mn, and Zn obtained using the simplified methods with those obtained using the reference method RM1.

The direct analysis of the untreated BRJ5 (SM1), as well as its sample solutions prepared by the successive dilutions with water (SM2-SM4), was established to be useless. Unfortunately, these alternative sample preparation procedures brought unsatisfactory results for Cu, Fe, Mn, and Zn as compared to the reference concentrations of these metals. In all cases, the absolute t_calculated_ values of the applied test were higher than its critical value (see the t_calculated_ values for the results achieved with the simplified methods SM1–SM4 in Table 1).

Because the increased dilutions of the samples did not result in lessening the biases between the determined concentrations of Cu, Fe, Mn, and Zn and the reference concentrations of these metals, it was concluded that the main source of the systematic error was certainly not related to the transport interferences caused by the eventual differences in the nebulization efficiency. Most likely, the element-specific interferences, related to the presence of the vast organic concomitants in the prepared sample solutions, were the reason for the hindered atomization of the Cu, Fe, Mn, and Zn bound species [19]. Hence, it was decided to add HNO_3_ in the reasonable amounts to the prepared sample solutions to enhance the in situ decomposition of the coexisting organic species in the flame and/or the release of Cu, Fe, Mn, and Zn from the bound forms into their simpler and uniform species, ensuring their efficient atomization.

Indeed, due to the relatively high temperature of the fuel-lean air-acetylene flame used in the present work and its low burning velocity, it was possible to establish suitable dilution/acidification conditions that provided reliable results for Cu, Fe, Mn, and Zn in the BRJ5 as compared to the results obtained with the reference method (RM1). Accordingly, it was found that the undiluted (SM10) or two-fold diluted (SM9) samples of the BRJ5, but acidified with HNO_3_ to its final concentration of 1 mol L^−1^, allowed to simultaneously determine the concentrations of Cu, Fe, Mn, and Zn that were statistically identical with those measured with the reference method (RM1). In this case, the absolute t_calculated_ values of the t-test were lower than its critical value (see Table 1).

The second mentioned alternative sample preparation procedure (SM9) was decided to be used in all further experiments. With this procedure (two-fold dilution along with acidification with HNO_3_ to 1 mol L^−1^), various BRJs were analyzed by FAAS on the content of Cu, Fe, Mn, and Zn with no prior wet open- or closed-vessel digestion in the concentrated oxidizing reagents. The selected sample preparation procedure reduced the amount of 14 mol L^−1^ (concentrated) HNO_3_ used (from 7 mL for wet digestion to 1.4 mL for acidification), lasted less than 15 min (instead of two hours and 15 min for wet digestion), while the electricity consumption related to the resistive heating of the samples (1.5 kWh per one sample) in a heating/digestion block was completely needless in this case.

Considering the outcomes of the F- and t-tests used, the concentrations of Cu, Fe, Mn, and Zn determined with the proposed method were as precise and accurate as those determined with the reference method (RM1). To confirm the reliability of the proposed simplified method (SM9), four additional BRJs, differing in the content of juice and other ingredients (BRJ2, BRJ8, BRJ11) or coming from another batch (BRJ5), were also selected and analyzed for the comparison. The analytical samples of these BRJs were wet digested with concentrated HNO_3_ in the digestion block to get the reference concentrations of Cu, Fe, Mn, and Zn and prepared in parallel by using the proposed alternative sample preparation procedure. Both sets of the resulting sample solutions were subjected to the analysis by FAAS and, in turn, the determined concentrations of Cu, Fe, Mn, and Zn were statistically compared using the two-sample t-test (at α = 0.05, df = 6). The results of this analysis are given in Table 2.

As before, the assumption about the same variance of both distributions was verified due to the outcomes of the F-test. Since the F_calculated_ values were lower than the critical value of this test, the usage of the t-test was justified. In reference to the outcomes of the abovementioned test, it was ascertained that the true results were achieved with the proposed simplified method (SM9) as compared to the results achieved with the reference method (RM1). The differences between the average concentrations of Cu, Fe, Mn, and Zn obtained with the compared methods were statistically insignificant because, for all the samples, the absolute t_calculated_ values of the applied test were lower than its critical value (see Table 2).

### 2.3. Analytical Performance and Application of the Simplified Analytical Method

The following figures of merit were assessed to characterize the simplified method of the analysis of BRJs: The limits of detection (LODs) of Cu, Fe, Mn, and Zn, the limits of quantification (LOQs) of these metals, the precision (in reference to the repeatability of the measurements, expressed as the RSD), and the trueness (in reference to the comparison of the results with the reference values, expressed as the relative error, ER). The recovery of Cu, Fe, Mn, and Zn at three different levels of the fortification were also studied as the mean of assessing the trueness of the results.

Accordingly, ten sample solutions of BRJ5 were prepared and analyzed in a series along with the respective blank solutions. The method LODs of Cu, Fe, Mn, and Zn were assessed as to their concentrations corresponding to the absorbances equal to 3 × SD_blank_, where the SD_blank_ was the standard deviation of the absorbance signals acquired for ten subsequent measurements of the blank solution (a 1 mol L^−1^ solution). These LODs were 0.014 mg L^−1^ for Cu, 0.044 mg L^−1^ for Fe, 0.010 mg L^−1^ for Mn, and 0.012 mg L^−1^ for Zn. These values of the LODs of Cu, Fe, Mn, and Zn were of the same order as the respective instrumental LODs. They were not much improved, since no pre-concentration was included at the stage of the sample preparation. Certainly, they were two to three times better than the LODs assessed for FAAS combined with wet digestion, for which the SD_blank_ values were higher in magnitude. The respective LOQs (as 10×SD_blank_) were equal to 0.050 mg L^−1^ (Cu), 0.14 mg L^−1^ (Fe), 0.036 mg L^−1^ (Mn), and 0.038 mg L^−1^ (Zn). The repeatability was within the range of 2%−3%, including 2.9% for Cu, 2.2% for Fe, 2.0% for Mn, and 3.0% for Zn. The trueness was better than 6% because the REs were −1.2% (Cu), +5.8% (Fe), +1.9% (Mn), and −4.1% (Zn). The recoveries of Cu, Mn, and Zn (added to the BRJ5 at the level of 1.0, 2.0 and 4.0 mg L^−1^) were quantitative. Their average values for all three levels of the fortification were changed in the following ranges: From 96.7% ± 1.4% to 99.6% ± 2.1% for Cu, from 98.2% ± 1.8% to 102.1% ± 2.2% for Fe, from 99.7% ± 1.0% to 102.0%± 1.9% for Mn, and from 99.5% ± 1.1% to 101.3% ± 1.5% for Zn. All these figures of merit indicated that the simplified method of analysis proposed in the present study provided precise and true results for Cu, Fe, Mn, and Zn determined in the BRJs.

The element analysis of the BRJs was infrequently reported in the literature. Domagala-Swiatkiewicz and Gastol [1] lately used the closed-vessel microwave-assisted wet digestion in concentrated HNO_3_ prior to the determination of the major (Ca, K, Mg, P, and S), minor (B, Cu, Fe, Mn, Sr, and Zn), and trace elements (Cd, Ni, and Pb) by ICP OES. On the other hand, Wruss et al. [4] proposed a 10-fold dilution of the samples using 1 mol L^−1^ HNO_3_ for the measurements of the concentrations of Cu, Fe, Mn, Ni, and Zn also by ICP OES. Unfortunately, both methods were not shown at all to be validated in the cited works. The present work presented for the first time the simplified and validated method for the element analysis of the BRJs by FAAS, where the treatment of the samples was just their two-fold dilution along with their acidification with HNO_3_ to 1 mol L^−1^. Considering all the samples (BRJ1–BRJ12) analyzed using the proposed simplified method, it was found that the concentrations of Cu, Fe, Mn, and Zn were quite heterogeneous. The mean concentrations of these metals were 0.158 mg L^−1^ (Cu), 1.91 mg L^−1^ (Fe), 2.63 mg L^−1^ (Mn), and 0.841 mg L^−1^ (Zn), however, the coefficients of variation (CVs) for the whole group of BRJs were quite high, i.e., 110% for Cu, 100% for Fe, 78% for Mn, and 110% for Zn. It was likely related with the fact that certain samples (BRJ7−BR12) contained some additional ingredients. When the latter samples were excluded from the statistical evaluation, and the samples containing only beetroot juice or its concentrate were considered (BRJ1−BRJ6), the respective CVs were about two-fold lower, i.e., 60% (Cu), 50% (Fe), 46% (Mn), and 67% (Zn). Considering the concentration ranges determined in the case of BRJ1−BRJ6, they well corresponded to these reported by Wruss and co-workers [4], who established the following values for eight freshly prepared BRJs: 0.022−0.392 mg L^−1^ for Cu (with the mean of 0.210 mg L^−1^ and the CV of 66%), 0.321−4.03 mg L^−1^ for Fe (with the mean of 2.21 mg L^−1^ and the CV of 52%), 0.352−1.20 mg L^−1^ for Mn (with the mean of 0.833 mg L^−1^ and the CV of 38%), and 0.105−2.20 mg L^−1^ for Zn (with the mean of 1.13 mg L^−1^ and the CV of 59%).

### 2.4. Sorption and Desorption Properties of the LC-18 and LC-SCX SPE Tubes

Initially, the sorption and desorption properties of the reversed-phase hydrophobic LC-18 and strong cation exchange LC-SCX SPE tubes were verified. At first, 10.0 mL of the solutions containing 0.2 mg L^−1^ of Cu, 1.5 mg L^−1^ of Fe, 2.5 mg L^−1^ of Mn, and 1.0 mg L^−1^ of Zn (pH 4.5, 5.0 and 5.5), with or without 500 mg L^−1^ of oxalic acid, were passed at 1 mL min^−1^ through the LC-18 SPE tubes. The respective effluents were collected and subjected to the analysis by FAAS on the content of Cu, Fe, Mn, and Zn. It was established that none of the studied metals was retained by the LC-18 SPE tubes in these conditions because they were completely recovered from the collected effluents, i.e., 99.2%−102.0% of Cu, 100.0–101.3% of Fe, 99.4–100.5% of Mn, and 99.7–101.0% of Zn. This pointed out that there was no concern that the hydrophobic fraction (HF) of Cu, Fe, Mn, and Zn, assessed using the reversed-phase LC-18 SPE tubes, could be overestimated due to the retention of the simple ions of Cu, Fe, Mn, and Zn or their complexes with low molecular weight (LMW) compounds like oxalic acid. In the next turn, 10.0 mL of the solutions containing 0.2 mg L^−1^ of Cu, 1.5 mg L^−1^ of Fe, 2.5 mg L^−1^ of Mn, and 1.0 mg L^−1^ of Zn (pH 4.5, 5.0 and 5.5), along with 500 mg L^−1^ of tannin added, were used to find a suitable eluent for the recovery of the Cu, Fe, Mn, and Zn species associated with the moderate and high molecular weight (MMW, HMW) compounds from the LC-18 SPE tubes. The mentioned mixed solutions were passed at 1 mL min^−1^ through the LC-18 tubes and the respective 10.0-mL effluents were collected. They were analyzed by FAAS to determine the amounts of the metal−tannin complexes retained by the LC-18 SPE tubes. To elute these complexes, the SPE tubes were rinsed with 5.0 mL of the tested 1, 2, and 3 mol L^−1^ HNO_3_ solutions, and next, with 5.0 mL of water, both also passed at 1 mL min^−1^. The respective 10.0-mL portions of the eluates were collected and analyzed by FAAS. Considering the amounts of the metal−tannin complexes formed at pH 5.5, which were the highest as shown by their sorption behavior on the LC-18 SPE tubes, it was found that the 2 and 3 mol L^−1^ HNO_3_ solutions gave the quantitative recoveries of Cu, Fe, Mn, and Zn. The average values (n = 3, ±SD) of the desorption efficiencies established using the 2 mol L^−1^ HNO_3_ solution were 98.8% ± 1.6% (Cu), 99.3% ± 1.2% (Fe), 98.9% ± 1.5% (Mn), and 99.8% ± 2.1% (Zn). The corresponding quantitative desorption efficiencies were achieved in the case of the 3 mol L^−1^ HNO_3_ solution, i.e., 99.0% ± 2.3% (Cu), 99.2% ± 1.4% (Fe), 100.1% ± 1.7% (Mn), and 99.7% ± 2.6% (Zn).

In the case of the LC-SCX SPE tubes, it was verified at first that Cu, Fe, Mn, and Zn were quantitatively retained from their mixed solutions, i.e., 0.2 mg L^−1^ of Cu, 1.5 mg L^−1^ of Fe, 2.5 mg L^−1^ of Mn, and 1.0 mg L^−1^ of Zn, pH 4.5, 5.0, and 5.5, when passing these solution through the tubes at 1 mL min^−1^. In all the cases, the concentrations of the studied metals in the collected effluents were below their respective LODs. Next, using the mixed solution buffered to pH 5.5, a suitable eluent for the recovery of Cu, Fe, Mn, and Zn from the LC-SCX SPE tubes was also selected amongst the 1, 2 and 3 mol L^−1^ HNO_3_ solutions. As before, the SPE tubes were rinsed with 5.0 mL of the tested HNO_3_ solutions, followed by 5.0 mL of water, both passed at 1 mL min^−1^. The respective 10.0-mL portions of the eluates were collected and analyzed by FAAS to evaluate the desorption efficiencies. Again, the 2 and 3 mol L^−1^ HNO_3_ solutions produced the quantitative recoveries of Cu, Fe, Mn, and Zn from the LC-SCX SPE tubes, i.e., 98.5–101.2% for Cu, 99.3–100.6% for Fe, 97.8–102.1% for Mn, and 98.0–103.1% for Zn.

### 2.5. Chemical Fractionation of Cu, Fe, Mn, and Zn in the Beetroot Juices

The methodology used in the present study for the chemical fractionation by the two-column SPE enabled to separate three operationally defined fractions of Cu, Fe, Mn, and Zn species, i.e., the HF, the RF, and the CF. A similar concept that based on the SPE with the mechanisms of the retention of the species by the reversed-phase followed by the cation exchange was recently used for a broad classification of the possible species of Cu and Fe in wine [19,20,21]. It enabled us to gain a valuable insight into the speciation of these metals and helped to understand the manner by which they were associated with different organic matrix components.

**Figure 1:**
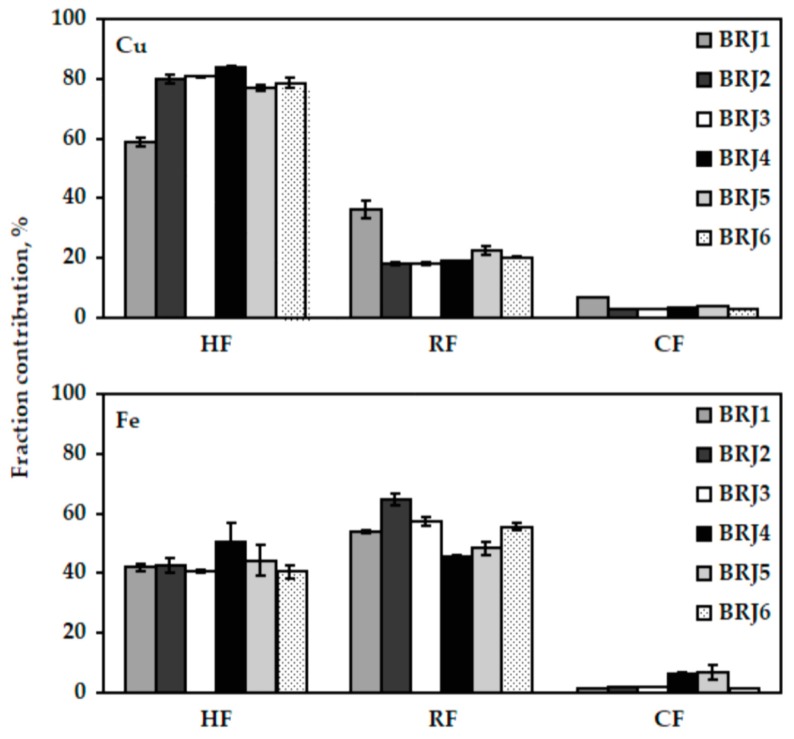
The contributions of the chemical fractions of the Cu and Fe species, i.e., the hydrophobic (HF), the residual (RF) and the cationic (CF), determined in the selected beetroot juices (BRJ1−BRJ6) using the two-column solid-phase extraction (SPE) with the LC-18 and LC-SCX tubes.

**Figure 2:**
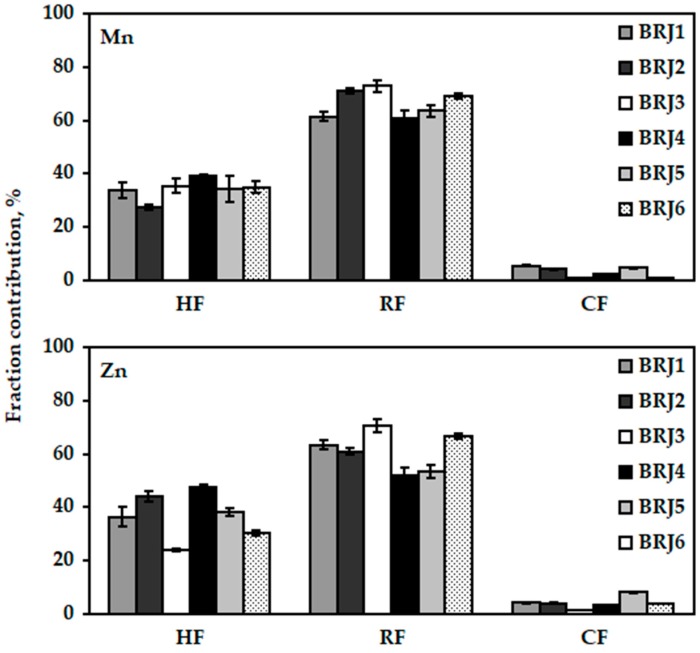
The contributions of the chemical fractions of the Mn and Zn species, i.e., the hydrophobic (HF), the residual (RF) and the cationic (CF), determined in the selected beetroot juices (BRJ1−BRJ6) using the two-column solid-phase extraction (SPE) with the LC-18 and LC-SCX tubes.

The chemical fractionation of Cu, Fe, Mn, and Zn with the aid of the two-column SPE (the LC-18 tubes followed by the LC-SCX tubes) was carried out for the samples BRJ1−BRJ6, which were declared to contain the highest amounts of juice. The results of this analysis are given in Figure 1 for Cu and Fe and Figure 2 for Mn, and Zn. In addition, they are given in the Supplementary Materials (Tables S1 and S2). Surprisingly, the species of all the studied metals were distributed amongst the HF and the RF, pointing out that Cu, Fe, Mn, and Zn were highly complexed by the components of the BRJ matrix. While the exact components of BRJs responsible for the speciation of Cu, Fe, Mn, and Zn were not certain, it was presumed that the HF was likely associated with the complexes of these metals with betalains, i.e., betacyanins and betaxanthins, which are recognized to bind the ions of many metals, including Cu and Fe [4,5]. Other hydrophobic macromolecules belonging to this fraction could be the complexes of the studied metals with the flavonoids like betagarin, betavulgarin, cochiophilin A and dihydroisorhamnetin, these compounds are also recognized to interact with the ions of different metals [4,5]. Considering that the concentrations of the abovementioned pigments and flavonoids in the BRJs are higher than 1000 mg L^−1^ [4,22], it was not surprising that the contributions of the HF of the studied metals were quite high, particularly in the case of Cu it was on average 76% (the CV of 12%), while for Fe it was on average 44% (the CV of 8.7%). The contributions of the HF of Mn and Zn were correspondingly 34% (the CV of 11%) and 37% (the CV of 23%).

The contributions of the RF of Cu, Fe, Mn, and Zn, which likely contained the negatively charged or neutral complexes of these metals, not sufficiently hydrophobic to be retained by the LC-18 SPE tubes, were even higher than those established for the HF. Accordingly, the RF could include the complexes of the studied metals with the phenolic acids, e.g., caffeic, coumaric, ferulic, gallic, syringic, vanillic, whose concentrations normally range from 30 to 40 mg L^−1^ [4,5,23], as well as other carboxylic acids, particularly oxalic acid, whose concentration varies from 300 to 500 mg L^−1^ [4]. The bound forms of the studied metals with the abovementioned compounds contributed to the RF on average with 22% in the case of Cu (the CV of 32%), 54% in the case of Fe (the CV of 12%), 66% in the case of Mn (the CV of 7.7%), and 61% in the case of Zn (the CV of 12%).

The contributions of the CF of the studied metals were very low, i.e., <4% (Cu), <3% (Fe), ~3% (Mn), and ~4% (Zn). This indicated that Cu, Fe, Mn, and Zn in the BRJ matrix hardly formed stable cationic complexes or such that were labile enough to be classified as cationic after the treatment with the LC-SCX SPE tubes. Hence, it was evident that the BRJs possessed a quite high complexing capacity toward the Cu, Fe, Mn, and Zn ions.

The sums of the HF, RF, and CF contributions achieved for Cu, Fe, Mn, and Zn were quantitative and changed within the following ranges: from <101.0% to <105.7% for Cu, from <97.5% to <109.2% for Fe, from 100.9% to 108.9% for Mn, and from 96.2% to 109.3% for Zn. The SD values of the established fraction contributions were within the following ranges: 0.3–2.8% for Cu, 0.3–6.2% for Fe, 0.1–4.9% for Mn, and 0.1–3.5% for Zn. Both measures were good and indicated the reliability of the results obtained with the proposed chemical fractionation procedure for Cu, Fe, Mn, and Zn in BRJs. It was additionally verified that the contributions of the HF determined using two different approaches, i.e., non-elution and elution, were quite comparable (see Tables S1 and S2).

Due to the presence of the negatively charged and/or neutral species of the studied metals with the LMW carboxylic acids and phenolic acids, which inhibit the absorption of the metals to a lower degree as compared to the polyphenols [24], it was presumed that the contribution of the RF could roughly estimate the extent of the bioavailability of Cu, Fe, Mn, and Zn from BRJs, however, this should mostly be verified by an in vitro study.

## 3. Materials and Methods

### 3.1. Instrumentation

The total concentrations of Cu, Fe, Mn, and Zn were measured using a simple-in-use PerkinElmer FAAS instrument, model 1100B. It was equipped with a 10-cm Ti burner to sustain a fuel-lean air-acetylene flame as an atomization cell. The solutions of the standards and samples were introduced into the air-acetylene flame through a sample introduction unit that was integrated with the burner head. It comprised a mixing chamber coated with an inert, wettable plastic and an end cap/siphon interlock assembly, both for the proper drainage of the excess solutions, and a stainless steel body nebulizer with a Pt/Ir capillary for the aspiration and nebulization of the solutions. A flow spoiler was inserted into the mixing chamber to remove the large droplets from the aerosol stream. The Lumina single-element hollow cathode lamps from PerkinElmer were used as the line radiation sources. Additionally, a Lumina deuterium lamp was applied for the background correction originating from any non-specific radiation. All the parameters recommended by the instrument manufacturer were used for its operation (see Table 3).

For the time-average integration of the absorbance signals, a “hold” mode was used with three read cycles and an integration time of one second for each one. In this signal processing, ten signal readings were carried out within 0.1-s intervals and next, integrated into each measurement cycle. Finally, the integrated signals from three cycles were averaged. Four solutions of the mixed (Cu, Fe, Mn, and Zn) standards were used to calibrate the FAAS instrument. They covered the concentration range from 0.05 to 2 mg L^−1^.

Additionally, an ICP OES instrument, model Agilent 720, with a horizontal plasma layout and the axial viewing of the plasma was applied for the reference analyses. It was equipped with a high-resolution Echelle-type polychromator and a Vistachip II solid-state charge-transfer device detector. The sample introduction system included an Agilent glass single-pass cyclonic spray chamber with an Agilent concentric OneNeb nebulizer. All settings recommended by the instrument manufacturer were used to operate it during the measurements (see Table 3). For the quantification of the total concentrations of Cu, Fe, Mn, and Zn, the four-point calibration curves were acquired using the solutions of their mixed standards, covering the concentration range within 0.01–2 mg L^−1^.

For wet digestion of the samples of BRJs, an SCP Science (Champlain, NY, USA) digestion block DigiPREP Jr. with a keypad controller to control and monitor the actual temperature was used. Graduated polypropylene (PP) 50-mL DigiTubes with polyethylene (PE) screw caps and PP ribbed watch glasses were applied as the digestion vessels.

### 3.2. Reagents and Materials

The analytical grade reagents and materials were used during the digestion of the samples of the BRJs in the digestion block and the extraction of the Cu, Fe, Mn, and Zn species by the two-column SPE. Re-distilled water was provided by a re-distiller REL-5 (Polna, Poland). The TraceCERT stock solutions (1000 mg L^−1^) of Cu, Fe, Mn, and Zn were supplied by Merck (Poland) and used to prepare the solutions of the mixed standards for the calibration of the FAAS and ICP OES instruments. Tannin and oxalic acid were purchased from Avantor Performance Materials (Poland). A Merck ACS grade solution of 65% HNO_3_ was used for wet digestion of the samples of the BRJs as well as the precondition and elution procedures related with the two-column SPE.

Supelclean LC-18 and LC-SCX SPE tubes were also supplied by Merck. Both kinds of the SPE tubes had PE frits (20 μ porosity) and the PP hardware. They were filled with the 500 mg portions of the irregularly shaped silica gel base material (particle size 40–45 μm), being monomerically bonded with the octadecyl groups in the case of LC-18 (for the reversed-phase retention mode of the Cu, Fe, Mn, and Zn species) or chemically modified with the sulfonic acid groups in the case of LC-SCX (for the cation exchange retention mode of the Cu, Fe, Mn, and Zn species). The beds of the LC-18 SPE tubes were washed with 5 mL of methanol and then with 10 mL of water, while the LC-SCX tubes were washed with 5 mL of a 2 mol L^−1^ HNO_3_ solution and then with 10 mL of water. The preconditioning reagents and water were passed through the tubes at a flow rate of 1 mL min^−1^ using a peristaltic pump.

### 3.3. Samples of Beetroot Juices

Several commercially available BRJs were used for this study, i.e., for the elemental analysis (Cu, Fe, Mn, and Zn) and the chemical fractionation of the given metals. The products that were selected for the present study differed as to the content of juice. Accordingly, there were juices with high amounts of freshly processed juice or its concentrate, i.e., 100% (BRJ1, BRJ2, and BRJ3), 99% (BRJ4, BRJ5), 97% (BRJ6), 87% (BRJ7 with the admixture of apple juice) and 80% (BRJ8 with the admixture of apple juice). There were also the products that contained lower amounts of juice and some additional ingredients, i.e., 83% (BRJ9), 69% (BRJ10), 60% (BRJ11) and 59% (BRJ12). In the case of each BRJ product, up to ten bottles/containers of a given BRJ were randomly taken from a store batch and their content was mixed together in a laboratory to prepare a gross sample. The volume of the latter gross sample was reduced to about 200 mL, and the resultant laboratory sample was placed in a clean screw-capped container and kept in a refrigerator (4 °C) prior to the elemental analysis and the chemical fractionation. The analytical samples were immediately sampled from the laboratory sample that were initially let to warm up to the room temperature and well-mixed by shaking.

### 3.4. Sample Preparation Procedures

#### 3.4.1. Wet Digestion

For wet digestion of the selected BRJs (BRJ2, BRJ5, BRJ8, and BRJ11), their 10.0-g analytical samples were placed in 50-mL DigiTUBES and treated with 5.0 mL of a 65% HNO_3_ solution. The digestion tubes were covered with the PP watch glasses and put into a DigiPREP Jr digestion block. The temperature of the digestion process was set at 130 °C and kept as such for two hours so as to reduce the volumes of the digests to less than 1 mL. After cooling, the resulting digests were treated again with 2.0 mL of a 65% HNO_3_ solution and heated (this time at 110 °C) for one hour until their volumes were reduced to less than 1 mL. Finally, the cooled digests were diluted with water to 20.0 g to reconstitute sample solutions. The sample solutions were finally filtered through 0.45 μm Simplepure PES-based syringe filters (Chemland, Poland). The resulting filtrates were subjected to the elemental analysis by FAAS and ICP OES against the simple solutions of the mixed Cu, Fe, Mn, and Zn standards. For each BRJ taken for wet digestion with concentrated HNO_3_, four analytical samples were taken from the laboratory sample and independently prepared following the procedure given above. The appropriate procedural blanks were also prepared using water instead of the samples. The final concentrations (in mg L^−1^) of total Cu, Fe, Mn, and Zn (C_total_) in the BRJs were blank-corrected and given as the average values along with the SDs.

#### 3.4.2. Non-digestive Sample Preparation

A rapid and simple sample preparation procedure that completely avoided wet digestion with concentrated HNO_3_ at the elevated temperature was also applied. To prepare the sample solutions of BRJs prior to the FAAS analysis on the content of Cu, Fe, Mn, and Zn, the 10.0-g samples were two-fold diluted with water and acidified with concentrated HNO_3_ so that its final concentration in the resulting sample solutions was 1 mol L_-1_. The resulting sample solutions were directly aspirated into a spectrophotometer for the determination of the C_total_ of Cu, Fe, Mn, and Zn versus the simple solutions of the mixed Cu, Fe, Mn, and Zn standards. For each BRJ, four sample solutions were prepared and analyzed along with the solutions of the respective procedural blanks (1 mol L^−1^ HNO_3_). If a given BRJ contained some naturally occurring precipitates, it was sonicated for 30 min at room temperature and filtered through a dense cellulose filter (Munktell Filtrak, Germany). The final results (in mg L^−1^) on the total content (C_total_) of Cu, Fe, Mn, and Zn in the BRJs were blank-corrected and given as the average values along with the SDs.

#### 3.4.3. Two-column Solid-Phase Extraction

**Figure 3:**
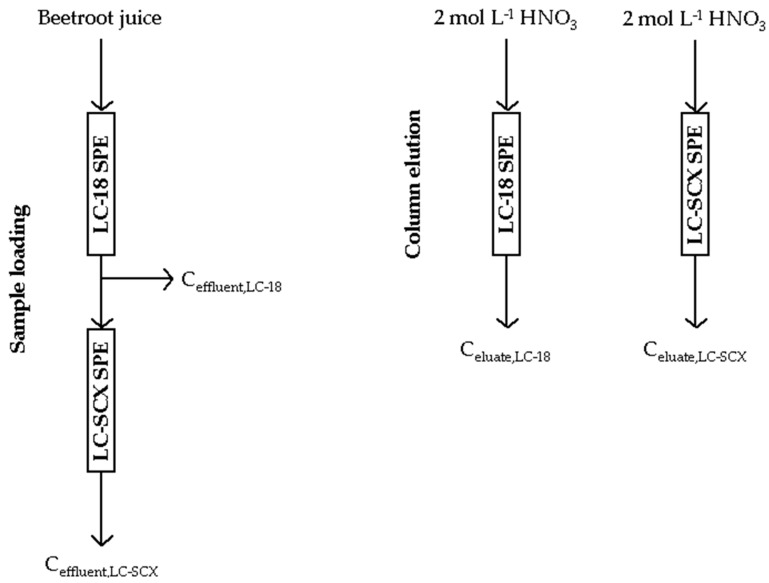
A scheme of the two-column solid-phase extraction (SPE) approach for the chemical fractionation of Cu, Fe, Mn, and Zn in the samples of beetroot juices.

Supelclean LC SPE tubes were used for the chemical fractionation of Cu, Fe, Mn, and Zn in the selected BRJs (BRJ1–BRJ6). A scheme of the two-column SPE procedure used for this purpose is given in Figure 3. At first, the 5.0-mL portions of the BRJs were passed at a flow rate of 1 mL min^−1^ through the LC-18 SPE tubes to retain the hydrophobic species of Cu, Fe, Mn, and Zn, likely associated with the presence of the stable complexes of these metals with the HMW and MMW compounds. The effluents of the LC-18 SPE tubes were collected and half of their portions (~2.5 mL) were saved for the determination of the concentration of Cu, Fe, Mn, and Zn species that were not retained by the LC-18 SPE tubes (C_effluent,LC-18_). Knowing the C_total_ of Cu, Fe, Mn, and Zn in the analyzed BRJs, the concentration of the Cu, Fe, Mn, and Zn species in the HF retained on the LC-18 SPE tubes was assessed by the following subtraction: C_total_-C_effluent,LC-18_. The remaining part of the effluents was successively passed at the same flow rate through the LC-SCX SPE tubes to retain the cationic species of Cu, Fe, Mn, and Zn. The effluents of these SPE tubes were collected and subjected to the analysis on the concentration of Cu, Fe, Mn, and Zn species that were not retained by both LC-18 and LC-SCX SPE tubes (C_effluent,LC-SCX_). The latter species could simply be the residual LMW species, neutral or anionic in reference to the overall charge, and contributed to the RF of the studied metals. In both mentioned cases, a rapid and simple sample preparation procedure was used to prepare the collected LC-18 and LC-SCX effluents before their analysis by FAAS versus the simple solutions of the mixed Cu, Fe, Mn, and Zn standards, i.e., they were two-fold diluted and acidified with HNO_3_ to 1 mol L^−1^. Finally, both kinds of the SPE tubes were split out, individually washed with water (10.0 mL), and then rinsed at 1 mL min^−1^ with 5.0 mL of a 2-mol L^−1^ HNO_3_ solution, followed by 5.0 mL of water. The respective eluates (10.0 mL) from both kinds of SPE tubes were collected and analyzed by FAAS for the concentration of Cu, Fe, Mn, and Zn retained by LC-18 (C_eluate,LC-18_) and LC-SCX (C_eluate,LC-SCX_) sorbents, respectively. These eluates were not specially treated prior to the FAAS analysis, and the simple solutions of the mixed Cu, Fe, Mn, and Zn standards were used for the quantification. In this way, the concentrations of Cu, Fe, Mn, and Zn in the HF and in the CF, being the simple ions of Cu, Fe, Mn, and Zn along with their stable cationic complexes with the LMW compounds and/or the labile species of these metals, were determined. The use of the non-elution and elution approaches to assess the concentration of Cu, Fe, Mn, and Zn in the HF allowed to compare these two procedures.

The appropriate procedural blanks, considering the steps of the sample loading and the metal species elution, were also prepared by repeating the whole procedure for both kinds of the SPE tubes but using the 5.0 mL portions of water adjusted to the pH 5.5 (the pH of the BRJ1-BRJ6 was in the range of 4.3–5.7). All the concentrations of Cu, Fe, Mn, and Zn determined in the effluents and eluates collected for the BRJ1–BR6 were used to calculate the percentage contributions of the fractions of Cu, Fe, Mn, and Zn, i.e., %HF = 100% × [(C_total_-C_effluent,LC-18_)/C_total_] and for the comparison as 100% × [C_eluate,LC-18_/C_total_], %RF = 100% × [C_effluent,LC-SCX_/C_total_], and %CF = 100% × [2 × C_eluate,LC-SCX_/C_total_]. For each BRJ analyzed, the chemical fractionation procedure was repeated twice and the respective fraction contributions (in %) were calculated as the average values along with the SDs. The results achieved for the procedural blanks were considered.

## 4. Conclusions

The present work reported for the first time a simplified method for the determination of the total concentrations of Cu, Fe, Mn, and Zn in beetroot juices by FAAS, including a very simple and fast sample preparation prior to the spectrometric measurements. Completely omitting the high-temperature wet decomposition of the sample matrix in a digestion block, the proposed method saved time (the sample treatment was nine-time faster) and completely eliminated the money expenses linked with electricity consumption. In this way, the developed method considerably increased the sample throughput, and hence, it could successfully be used for the routine control of the quality and safety of the popular vegetable juices. It also eliminated the problems related with the possible contamination of the analyzed samples and/or the losses of the analytes due to wet digestion in the concentrated reagents. The method indeed appeared to be useful in the fast screening analysis of several commercially available beetroot juices. Its suitability was also verified during the chemical fraction of Cu, Fe, Mn, and Zn in the selected beetroot juices because it enabled to uncomplicatedly determine the concentrations of Cu, Fe, Mn, and Zn in the effluents resulting from the SPE treatment of the samples.

The mentioned chemical fractionation of beetroot juices, in which the species of Cu, Fe, Mn, and Zn were separated with the aid of the reversed-phase and then cation-exchange sorbents, revealed that all the studied metals were organically bound by the beetroot juice matrix components to a very high degree. Accordingly, the sums of the mean contributions of the hydrophobic and residual fractions were 98% (Cu, Fe, Zn) and 100% (Mn), pointing out that no labile or unbound forms of Cu, Fe, Mn, and Zn were present in the analyzed beetroot juices.

## Figures and Tables

**Table 1:** Comparison of the total concentrations of Cu, Fe, Mn, and Zn (in mg L^−1^) determined in the selected beetroot juice (BRJ5) using flame atomic absorption spectrometry (FAAS) combined with wet digestion with concentrated HNO_3_ in a digestion block (the reference method no. 1, RM1) and the alternative sample preparation procedures (the simplified methods, SM1-SM10). Inductively coupled plasma optical emission spectrometry (ICP OES) combined with wet digestion with concentrated HNO_3_ in a digestion block was used as the reference method no. 2 (RM2).

	Cu	Fe	Mn	Zn
*The reference methods—wet digestion with HNO_3_ followed by the FAAS (RM1) or ICP OES (RM2) analysis*				
RM1	0.117 (±2.6%)	2.27 (±2.6%)	2.70 (±2.4%)	0.598 (±2.4%)
	1.457^1^, −1.524^2^	2.761^1^, +1.457^2^	3.856^1^, −1.367^2^	1.737^1^, −0.795^2^
RM2	0.120 (±2.1%)	2.22 (±1.6%)	2.75 (±1.2%)	0.605 (±1.8%)
*The simplified methods—dilution with water followed by the FAAS analysis*				
SM1, 5-fold	<0.029	1.19 (±2.2%)	1.19 (±2.3%)	0.336 (±2.6%)
		1.841^3^, −33.006 ^4^	1.454^3^, −12.154 ^4^	1.554^3^, −31.688 ^4^
SM2, 3-fold	0.036 (±12.5%)	1.24 (±2.4%)	2.20 (±1.4%)	0.314 (±2.2%)
	3.189^3^, −30.103 ^4^	1.425^3^, −30.782 ^4^	1.148^3^, −13.708 ^4^	2.485^3^, −36.222 ^4^
SM3, 2-fold	0.064 (±5.0%)	1.20 (±2.9%)	2.18 (±1.5%)	0.364 (±4.3%)
	1.612^3^, −24.116 ^4^	1.042^3^, −30.893 ^4^	1.018^3^, −14.049 ^4^	2.066^3^, −22.162 ^4^
SM4, no dilution	0.082 (±2.5%)	1.14 (±1.8%)	2.06 (±2.4%)	0.360 (±2.3%)
	1.511^3^, −18.999 ^4^	2.996^3^, −36.311 ^4^	2.245^3^, −16.495 ^4^	1.730^3^, −30.905 ^4^
*The simplified methods—dilution with water along with the acidification with HNO_3_ to 0.5 mol L ^−1^ followed by the FAAS analysis*				
SM5, 3-fold	0.071 (±9.6%)	2.31 (±2.3%)	2.56 (±2.3%)	0.600 (±3.8%)
	7.316 ^3^, −12.372 ^4^	2.237^3^, +1.047^4^	3.184^3^, −2.980 ^4^	4.383^3^, +0.138^4^
SM6, 2-fold	0.109 (±4.8%)	2.34 (±3.1%)	2.33 (±1.2%)	0.629 (±3.8%)
	4.311^3^, −2.644 ^4^	4.171^3^, +1.492^4^	1.393^3^, −10.264 ^4^	4.817^3^, +2.228^4^
SM7, no dilution	0.111 (±4.0%)	2.21 (±1.2%)	2.15 (±1.6%)	0.650 (±1.2%)
	3.104^3^, −1.898^4^	1.794^3^, −1.559^4^	1.087^3^, −14.716 ^4^	1.949^3^, +6.516 ^4^
*The simplified methods—dilution with water along with the acidification with HNO_3_ to 1.0 mol L ^−1^ followed by the FAAS analysis*				
SM8, 3-fold	0.091 (±6.2%)	2.33 (±2.6%)	2.78 (±1.7%)	0.598 (±2.7%)
	5.013^3^, −7.950 ^4^	2.909^3^, +1.547^4^	2.051^3^, +2.162^4^	2.198^3^, +0.020^4^
SM9, 2-fold	0.115 (±3.5%)	2.30 (±2.4%)	2.77 (±1.9%)	0.594 (±2.3%)
	2.551^3^, −0.554^4^	2.415^3^, +0.796^4^	2.544^3^, +1.830^4^	1.574^3^, −0.451^4^
SM10, no dilution	0.124 (±4.0%)	2.33 (±1.7%)	2.71 (±1.3%)	0.596 (±1.6%)
	3.874^3^, +2.400^4^	1.244^3^, +1.866^4^	1.140^3^, +0.426^4^	1.304^3^, −0.226^4^

The average values (n = 4) with the relative standard deviations in brackets (±RSD). ^1^ The calculated values of the F-test (F_calculated_ at α = 0.05 and df_1_ = 3, df_2_ = 3) for the comparison of the standard deviation of the average concentrations of Cu, Fe, Mn, and Zn achieved using wet digestion in the digestion block followed by the analysis of the resulting sample solutions by FAAS (RM1) and ICP OES (RM2). The F_calculated_ values lower than the critical value of the test (F_critical_ = 5.391) are underlined. ^2^ The calculated values of the two-sample t-test (t_calculated_ at α = 0.05 and df = 6) for the comparison of the average concentrations of Cu, Fe, Mn, and Zn achieved using wet digestion in the digestion block followed by the analysis of the resulting sample solutions by FAAS (RM1) and ICP OES (RM2). The t_calculated_ values lower than the critical value of the test (t_critical_ = 2.447) are underlined. ^3^ The calculated values of the F-test (F_calculated_ at α = 0.05 and df_1_ = 3, df_2_ = 3) for the comparison of the standard deviation of the average concentrations of Cu, Fe, Mn, and Zn achieved using wet digestion in the digestion block followed by the analysis of the resulting sample solutions by FAAS (RM1) and the alternative sample preparation procedures followed by the analysis of the resulting sample solutions by FAAS (SM1-SM10). The F_calculated_ values lower than the critical value of the test (F_critical_ = 5.391) are underlined. ^4^ The calculated values of the two-sample t-test (t_calculated_ at α = 0.05 and df = 6) for the comparison of the average concentrations of Cu, Fe, Mn, and Zn achieved using wet digestion in the digestion block followed by the analysis of the resulting sample solutions by FAAS (RM1) and the alternative sample preparation procedures followed by the analysis of the resulting sample solutions by FAAS (SM1-SM10). The t_calculated_ values lower than the critical value of the test (t_critical_ = 2.447) are underlined.

**Table 2:** The total concentrations (in mg L^−1^) of Cu, Fe, Mn, and Zn in beetroot juices (BRJs) determined by flame atomic absorption spectrometry (FAAS) combined with the alternative sample preparation (the 2-fold dilution and the acidification with HNO_3_ to 1 mol L^−1^). For the selected beetroot juices (BRJ2, BRJ5, BRJ8, BRJ11), the concentrations of Cu, Fe, Mn, and Zn were also determined by the reference method RM1, i.e., the FAAS analysis of the sample solutions resulted from wet digestion with concentrated HNO_3_ in the digestion block.

Sample	Cu	Fe	Mn	Zn
BRJ1	0.091 (±7.1%)	0.880 (±4.1%)	0.421 (±3.5%)	0.233 (±6.6%)
BRJ2 ^1^	0.296 (±4.4%)	1.71 (±3.3%)	2.30 (±1.9%)	1.65 (±3.6%)
BRJ2	0.298 (±3.3%)	1.71 (±3.3%)	2.30 (±1.9%)	1.65 (±3.6%)
	1.754^2^, +0.245^3^	1.405^2^, +1.146^3^	1.254^2^, +0.912^3^	2.778^2^, −0.860^3^
BRJ3	0.135 (±3.4%)	1.06 (±4.0%)	3.75 (±3.2%)	0.811 (±4.2%)
BRJ4	0.089 (±7.3%)	2.45 (±1.4%)	3.48 (±2.4%)	0.915 (±2.4%)
BRJ5 ^1^	0.128 (±8.6%)	2.16 (±1.0%)	2.73 (±2.7%)	0.598 (±3.8%)
BRJ5	0.121 (±6.6%)	2.18 (±2.0%)	2.77 (±1.2%)	0.608 (±3.0%)
	1.900^2^, −1.030^3^	4.074^2^, +0.582^3^	4.917^2^, −0.935^3^	1.552^2^, +0.681^3^
BRJ6	0.085 (±8.3%)	0.617 (±1.8%)	2.80 (±1.6%)	0.321 (±3.5%)
BRJ7	0.062 (±10.1%)	1.85 (±1.4%)	2.81 (±1.3%)	0.309 (±5.2%)
BRJ8 ^1^	<0.010	0.214 (±4.7%)	0.451 (±3.8%)	0.076 (±5.3%)
BRJ8	<0.010	0.210 (±4.9%)	0.455 (±4.2%)	0.082 (±10.0%)
		1.074^2^, −0.558^3^	1.243^2^, +0.313^3^	4.144^2^, +1.313^3^
BRJ9	0.395 (±2.3%)	2.62 (±2.6%)	0.581 (±2.7%)	1.61 (±1.1%)
BRJ10	<0.015	0.885 (±3.8%)	2.91 (±1.5%)	0.169 (±3.2%)
BRJ11 ^1^	<0.010	0.963 (±1.6%)	1.34 (±2.2%)	0.168 (±4.9%)
BRJ11	<0.015	0.952 (±1.3%)	1.33 (±1.7%)	0.165 (±3.5%)
		1.550^2^, −1.131^3^	1.700^2^, −0.532^3^	2.032^2^, −0.598^3^
BRJ12	0.570 (±1.0%)	7.44 (±0.9%)	8.01 (±1.5%)	3.25 (±1.1%)
BRJ1–BRJ12				
Maximum	0.570	7.44	8.01	3.25
Minimum	<0.015	0.201	0.421	0.082
Mean	0.158	1.91	2.63	0.841
CV	110%	100%	78%	110%
RSD range	1.0–10.1%	0.9–4.9%	1.5–4.2%	1.1–10.0%
BRJ1–BRJ6				
Maximum	0.298	2.45	3.75	1.62
Minimum	0.085	0.617	0.421	0.233
Mean	0.137	1.49	2.58	0.751
CV	60%	50%	46%	67%

The average values (n = 4) with the relative standard deviations in brackets (±RSD). ^1^ The reference method RM1 was used. ^2^ The calculated values of the F-test (F_calculated_ at α = 0.05 and df_1_ = 3, df_2_ = 3) for the comparison of the standard deviation of the average concentrations of metals determined by using the simplified and reference methods. The F_calculated_ values lower than the critical value of the test (F_critical_ = 5.391) are underlined. ^3^ The calculated values of the two-sample t-test (t_calculated_ at α = 0.05 and df = 6) for the comparison of the average concentrations of metals determined by using the simplified and reference methods. The t_calculated_ values lower than the critical value of the test (t_critical_ = 2.447) are underlined. CV The coefficient of variation.

**Table 3:** Operating parameters used for flame atomic absorption spectrometry (FAAS) and inductively coupled plasma optical emission spectrometry (ICP OES).

	Cu	Fe	Mn	Zn
FAAS				
Acetylene flow rate, L min^−1^	2.0			
Airflow rate, L min^−1^	8.0			
Wavelength, nm	324.8	248.3	279.5	213.9
Bandpass, nm	0.7	0.2	0.2	0.7
Lamp current, mA	15	30	15	15
ICP OES				
Plasma Ar flow rate, L min^−1^	15			
Auxiliary Ar flow rate, L min^−1^	1.50			
Nebulizing Ar flow rate, L min^−1^	0.75			
Sample uptake rate, mL min^−1^	1.2			
Forward power, kW	1.2			
Stabilization time, s	15			
Integration time, s	1			
Wavelengths, mA	324.8	248.3	257.6	206.2
Background correction	“Fitted” mode			

## Supplementary Materials

The following are available online. Table S1: The contributions of the chemical fractions of the Cu and Fe species, i.e., the hydrophobic (HF), the residual (RF) and the cationic (CF), determined in the selected beetroot juices (BRJ1-BRJ6) using the tandem solid phase extraction (SPE) with the LC-18 and LC-SCX tubes; Table S2: The contributions of the chemical fractions of the Mn and Zn species, i.e., the hydrophobic (HF), the residual (RF) and the cationic (CF), determined in the selected beetroot juices (BRJ1-BRJ6) using the tandem solid phase extraction (SPE) with the LC-18 and LC-SCX tubes.

## References

[1] Domagala-Swiatkiewicz D., Gastol M. (2012). Comparative study on mineral content of organic and conventional carrot, celery and red beet juices. Acta Sci. Pol. Hort. Cult..

[2] Netherlands Ministry of Foreign Affairs, Centre for the Promotion of Imports from Developing Countries Exporting Vegetable Juices to Europe. https://www.cbi.eu/market-information/processed-fruit-vegetables-edible-nuts/vegetable-juices.

[3] Nahla T.K., Wisam S.U., Tariq N.M. (2018). Antioxidant activities of beetroot (*Beta vulgaris* L.) extracts. Pak. J. Nutr..

[4] Wruss J., Waldenberger G., Huemer S., Uygun P., Lanzerstorfer P., Muller U., Hoglinger O., Weghuber J. (2015). Compositional characteristics of commercial beetroot products and beetroot juice prepared from seven beetroot varieties grown in Upper Austria. J. Food Comp. Anal..

[5] Chhikara N., Kushwaha K., Sharma P., Gat Y., Panghal A. (2019). Bioactive compounds of beetroot and utilization in food processing industry: A critical review. Food Chem..

[6] Babagil A., Tasgin E., Nadaroglu H., Kaymak H.C. (2018). Antioxidant and antiradical activity of beetroot (*Beta vulgaris* L. var. conditiva Alef.) grown using different fertilizers. J. Chem..

[7] Singh J.P., Kaur A., Shevkani K., Singh N. (2016). Composition, bioactive compounds and antioxidant activity of common Indian fruits and vegetables. J. Food Sci. Technol..

[8] Petek M., Toth N., Pecina M., Karazija T., Herak Custic M. (2016). Macrominerals in red beetroot under organic and mineral fertilization. Agric. Conspec. Sci..

[9] Lisiewska Z., Kmiecik W., Gebczynski P. (2006). Effects of mineral content of different methods of preparing frozen root vegetables. Food Sci. Technol. Int..

[10] Pajevic S., Arsenov D., Nikolic N., Borisev M., Orcic D., Zupunski M., Mimica-Dukic N. (2018). Heavy metal accumulation in vegetable species and health risk assessment in Serbia. Environ. Monit. Assess..

[11] Petek M., Toth N., Pecina M., Lazarevic B., Palcic I., Herak Custic M. (2017). Status of Fe, Mn, and Zn in red beet due to fertilization and environment. J. Centr. Eur. Agric..

[12] Niziol-Lukaszewska Z., Gaweda M. (2016). Influence of cultivar on the content of selected minerals in red beetroots (*Beta vulgaris* L.). Folia. Hort..

[13] Rekowska E., Jurga-Szlempo B. (2011). Content of mineral components in toots of selected cultivars of beetroot. J. Elem..

[14] Szakova J., Havlik J., Valterova B., Tlustos P., Goessler W. (2010). The contents of risk elements, arsenic speciation, and possible interactions of elements and betalains in beetroot (*Beta vulgaris* L.) growing in contaminated soil. Centr. Eur. J. Biol..

[15] (1994). ISO Standard No. 5724-4:1994, Accuracy (Trueness and Precision) of Measurement Methods and Results. Part 4: Basic Methods for the Determination of the Trueness of a Standard Measurement Method.

[16] Pohl P., Dzimitrowicz A., Jamroz P., Greda K. (2018). HR-CS FAAS based method for direct determination of total concentrations of Ca, Fe, Mg and Mn in functional apple beverages and evaluation of contributions of the bioaccessible fraction of these elements by in vitro gastrointestinal digestion and chemical fractionation. Microchem. J..

[17] Miller J., Miller J.C. (2018). Statistics and Chemometrics for Analytical Chemistry.

[18] Welz B., Seiler H.G., Sigel A., Sigel H. (1994). Atomic absorption spectrometry. Handbook on Metals in Clinical and Analytical Chemistry.

[19] Welz B., Sperling M. (2008). Atomic Absorption Spectrometry.

[20] Kontoudakis N., Schmidtke L.M., Bekker M.Z., Smith M., Smith P.A., Scollary G.R., Wilkes E.N., Clark A.C. (2019). Analytical strategies for the measurement of different forms of Cu and Fe in wine: Comparison between approaches in relation to wine composition. Food Chem..

[21] Kontoudakis N., Smith M., Guo A., Smith P.A., Scollary G.R., Wilkes E.N., Clark A.C. (2017). The impact of wine components on fractionation of Cu and Fe in model wine system: Macromolecules, phenolic and sulfur compounds. Food Res. Int..

[22] Kontoudakis N., Guo A., Scollary G.R., Clark A.C. (2017). The impact of aging wine in high and low oxygen conditions on the fractionation of Cu and Fe in Chardonnay wine. Food Chem..

[23] Kazimierczak R., Silakiewicz A., Hallmann E., Srednicka-Tober D., Rembialkowska E. (2016). Chemical composition of selected beetroot juices in relation to beetroot production system and processing technology. Not. Bot. Horti. Agrobot..

[24] Drago S.R., Galanakis C. (2017). Minerals. Nutraceutical and Functional Food Components: Effects of Innovative Processing Techniques.

