# Supplementary material for: New Green Determination of Cu, Fe, Mn, and Zn in Beetroot Juices along with Their Chemical Fractionation by Solid-Phase Extraction
Source: Molecules. 2019 Oct 9;24(20):3645. doi: 10.3390/molecules24203645 (PMC6832732; doi:10.3390/molecules24203645)
Supplement: Supplementary file 1 [file molecules-24-03645-s001.pdf]

Table S1. The contributions of the chemical fractions of the Cu and Fe species, *i.e.*, the hydrophobic (HF), the residual (RF) and the cationic (CF), determined in the selected beetroot juices (BRJ1-BRJ6) using the tandem solid phase extraction (SPE) with the LC-18 and LC-SCX tubes.

Sample	Fraction contribution[1], %			
	HF	RF	CF	Sum[2]
		Cu		
BRJ1	58.9±1.4% (61.2±2.3%)[3]	36.3±2.8	<6.8[4]	<102.0
BRJ2	80.0±1.5% (78.9±1.8%)[3]	18.0±0.4	<3.0[4]	<101.0
BRJ3	80.7±0.3% (79.1±2.0%)[3]	18.2±0.4	<3.1[4]	<102.0
BRJ4	83.6±0.6% (83.0±1.7%)[3]	18.9±0.1	<3.2[4]	<105.7
BRJ5	76.9±1.1% (76.5±1.6%)[3]	22.4±1.5	<3.7[4]	<103.0
BRJ6	78.6±1.8% (78.9±2.4%)[3]	20.2±0.3	<3.0[4]	<101.8
Maximum	83.6 (83.0)	36.3		<105.7
Minimum	58.9 (61.2)	18.0		<101.0
Mean	76.5 (76.3)	22.3		
CV, %	11.6 (10.0)	31.5		
SD range, %	0.3-1.8 (1.6-2.4)	0.4-2.8		
		Fe		
BRJ1	42.0±1.2% (41.6±0.9%)[3]	53.9±0.7	<1.6[4]	<97.5
BRJ2	42.6±3.8% (43.8±2.4%)[3]	64.6±1.9	<2.0[4]	<109.2
BRJ3	40.8±0.4% (41.2±0.8%)[3]	57.5±1.4	<2.0[4]	<100.3
BRJ4	50.7±6.2% (52.6±2.5%)[3]	45.6±0.4	6.6±0.3	102.9
BRJ5	44.3±5.1% (44.8±2.1%)[3]	48.5±2.2	6.7±2.5	99.5
BRJ6	40.5±2.2% (38.6±1.6%)[3]	55.6±1.2	<1.6[4]	<97.7
Maximum	50.7 (52.6)	64.6	6.7	<105.7
Minimum	40.5 (38.6)	45.6	<1.6	<101.0
Mean	43.5 (43.8)	54.3	<3.4	
CV, %	8.7 (11.1)	12.4		
SD range, %	0.4-6.2 (0.8-2.5)	0.4-2.2	0.3-2.5	

[1] The average value (n=2) along with the standard deviation (SD).

[2] The sum of the fraction contributions (%HF+%RF+%CF).

[3] Assessed on the basis of the concentrations of the Cu and Fe species determined in the effluents of the LC-18 SPE tubes.

[4] The eluates of the LC-SCX SPE tubes contained the Cu and Fe species at the levels below their limits of detection (LODs).

CV The coefficient of variation.

Table S2. The contributions of the chemical fractions of the Mn and Zn species, *i.e.*, the hydrophobic (HF), the residual (RF) and the cationic (CF), determined in the selected beetroot juices (BRJ1-BRJ6) using the tandem solid phase extraction (SPE) with the LC-18 and LC-SCX tubes.

Sample	Fraction contribution[1], %			
	HF	RF	CF	Sum[2]
		Mn		
BRJ1	33.8±2.8% (31.2±1.7%)[3]	61.5±1.6	5.6±0.3	100.9
BRJ2	27.4±1.0% (28.1±2.1%)[3]	71.1±1.2	4.2±0.1	102.9
BRJ3	35.3±2.7% (35.1±2.5%)[3]	72.8±2.4	0.8±0.1	108.9
BRJ4	39.1±0.7% (41.3±1.2%)[3]	60.9±2.9	2.3±0.2	102.3
BRJ5	34.3±4.9% (33.7±2.9%)[3]	63.6±2.3	4.8±0.3	102.7
BRJ6	35.0±2.2% (34.5±1.6%)[3]	69.0±1.1	0.9±0.1	104.9
Maximum	39.1 (41.3)	72.8	5.6	108.9
Minimum	27.4 (28.1)	60.9	0.8	100.9
Mean	34.2 (34.0)	66.5	3.1	
CV, %	11.1 (13.0)	7.7	66.3	
SD range, %	0.7-4.9 (1.2-2.9)	1.1-2.9	0.1-0.3	
		Zn		
BRJ1	36.5±3.5% (38.2±3.0%)[3]	63.4±1.6	4.2±0.1	104.1
BRJ2	44.2±1.9% (45.4±0.9%)[3]	61.0±1.2	4.1±0.1	109.3
BRJ3	24.2±0.5% (22.3±1.2%)[3]	70.6±2.4	1.4±0.1	96.2
BRJ4	47.5±1.2% (46.8±1.8%)[3]	51.9±2.9	3.4±0.2	102.8
BRJ5	38.2±1.4% (35.4±1.5%)[3]	53.5±2.3	8.1±0.3	99.8
BRJ6	30.3±1.1% (32.0±2.8%)[3]	66.7±1.1	3.8±0.1	100.8
Maximum	47.5 (46.8)	70.6	8.1	109.3
Minimum	24.2 (22.3)	51.9	1.4	96.2
Mean	36.8 (36.7)	61.2	4.2	
CV, %	23.4 (24.7)	12.0	52.4	
SD range, %	0.5-3.5 (0.9-3.0)	1.2-2.9	0.1-0.3	

[1] The average value (n=2) along with the standard deviation (SD).

[2] The sum of the fraction contributions (%HF+%RF+%CF).

[3] Assessed on the basis of the concentrations of the Mn and Zn species determined in the effluents of the LC-18 SPE tubes.

CV. The coefficient of variation.
